# Operationalising Environmental DNA (eDNA) Detection of Major Malaria Vector Species in Ghana

**DOI:** 10.1111/1755-0998.70154

**Published:** 2026-07-28

**Authors:** Mojca Kristan, Yaw A. Afrane, Sophie Moss, Yaw Akuamoah‐Boateng, Abdul Rahim Mohammed Sabtiu, Judith Dzifa Azumah, Anisa Abdulai, Isaac Kwame Sraku, Bright Churchill Obeng, Karen L. Figueroa Chilito, Jo Lines, Sian E. Clarke, Taane G. Clark, Susana Campino, Louisa A. Messenger

**Affiliations:** ^1^ Faculty of Infectious Tropical Diseases London School of Hygiene and Tropical Medicine London UK; ^2^ Centre for Vector‐Borne Disease Research, Department of Medical Microbiology, Medical School University of Ghana Accra Ghana; ^3^ Faculty of Epidemiology and Population Health London School of Hygiene and Tropical Medicine London UK; ^4^ Department of Environmental and Global Health, School of Public Health University of Nevada Las Vegas Nevada USA; ^5^ Parasitology and Vector Biology (PARAVEC) Laboratory, School of Public Health, University of Nevada Las Vegas Nevada USA

**Keywords:** *A. gambiae* s.l., amplicon‐sequencing, breeding sites, environmental DNA (eDNA), Ghana, insecticide resistance, mosquito larvae, qPCR, species identification, vector surveillance

## Abstract

The global landscape of vector‐borne diseases (VBDs) is shifting, driven by climate change, urbanisation and widespread habitat modification, requiring novel approaches to surveillance, prevention and control. To improve our capacity to respond to real‐time emergent VBD threats, this study undertook cross‐sectional sampling of suspected mosquito breeding sites in Greater Accra and Western Region, Ghana, to assess the operational feasibility of environmental DNA (eDNA) detection of major malaria vector species. eDNA deposited by wild, immature *Anopheles gambiae* s.l., isolated from a 50 mL water sample, was detectable using both end‐point PCR and qPCR, achieving high levels of sensitivity (76.28%) and specificity (100%). Furthermore, we recovered PCR amplicons covering eight known insecticide resistance mutations from eDNA collected in natural breeding sites. This study represents the first proof‐of‐concept demonstrating that eDNA can be used for the simultaneous identification of *Anopheles* vectors and circulating insecticide resistance alleles directly from aquatic habitats in the community, including the first report of the mutation *gste2*‐F120L in Ghana. Our eDNA sampling methodology was designed for citizen scientists as end‐point users. We recommend fixed, single‐use filters for sampling 50 mL of water as a highly feasible, sensitive and specific, community‐led mosquito vector surveillance tool, which requires minimal training or technical infrastructure.

## 1 Introduction

The epidemiology of vector‐borne diseases (VBD) is undergoing unpredictable shifts driven by concomitant climate change, urbanisation, globalisation and widespread ecological disruptions (Thomson and Stanberry 2022; Rocklöv and Dubrow 2020). Rising temperatures, altered precipitation patterns and habitat modification are expanding the geographic range of key vector species, including mosquitoes, increasing the risk of dengue, chikungunya, Zika, yellow fever and malaria in previously non‐endemic regions (Salkeld et al. 2023; Paz 2024). Greater connectivity due to international travel and trade can accelerate the introduction and establishment of novel pathogens and vectors in naïve populations (Kulkarni 2016; Gubler et al. 2025). Rapid urbanisation and deforestation create permissive environments for vector proliferation, while changes in land use, population density and human behaviour significantly contribute to altered VBD transmission dynamics (Ortiz et al. 2021; Ferraguti et al. 2023; Estifanos et al. 2024). Together, these factors are reshaping the global landscape of VBD risk, requiring novel monitoring, prevention and control approaches.

The timely detection and mitigation of invasive mosquito species remains a major public health challenge, driven by ecological, behavioural and surveillance gaps. Small founder populations often escape routine detection, especially in regions with intermittent or insufficient surveillance programmes (Nguyen et al. 2023; Dorzaban et al. 2022). Cryptic behavioural adaptations, such as altered resting or feeding patterns, allow invasive and native mosquitoes to evade traditional trapping methods. Seasonal fluctuations further obscure their presence, enabling low‐level vector density to persist unnoticed until favourable environmental conditions elicit expansion (Nikookar et al. 2020). Misidentification of early specimens, particularly when their morphology closely resembles native species, compounds delays in notification. Critically, reliance on passive public reporting rather than proactive, systematic monitoring often means that new introductions are only discovered once populations are already entrenched (Ibañez‐Justicia et al. 2019). For example, in the past 30–40 years, *Aedes albopictus* has spread from its native home range in East Asia and islands of the western Pacific and Indian Ocean to colonise every continent, except Antarctica, where it is now responsible for focal dengue and Chikungunya outbreaks worldwide (Bonizzoni et al. 2013). More recently, *Anopheles stephensi*, a highly competent malaria vector, whose distribution until 2011 encompassed the Indian subcontinent, parts of South‐East Asia and the Arabian Peninsula, invaded the Horn of Africa, now threatening to reverse contemporary malaria gains across affected countries (Taylor et al. 2024; Tadesse et al. 2021; Emiru et al. 2023). Strengthening early warning systems and expanding monitoring initiatives are essential to prevent the continued spread of invasive vector species (Eritja et al. 2021; Lühken et al. 2023).

One potential method to increase efficient vector sampling capacity, to reveal less productive or entirely unknown breeding sites and therefore putative disease hotspots, may be to exploit environmental DNA (eDNA) detection. eDNA refers to genetic material sampled from the environment rather than the organism itself (Thomsen and Willerslev 2015). This technique can offer a non‐invasive, highly sensitive alternative for surveillance, particularly of low levels of target organisms or cryptic species found in complex environments (e.g., water, soil or air). This method requires no prior knowledge of target species morphology and is quick and easy to implement at the sampling stage, facilitating its integration into community citizen science initiatives (Garamszegi, Kurucz, et al. 2023; Garamszegi, Soltész, et al. 2023). eDNA detection has gained prominence in conservation biology, ecology and environmental management, facilitating species distribution tracking, assessments of ecosystem health and pathogen identification in aquatic and terrestrial environments. Furthermore, advances in next‐generation sequencing technologies and parallel depreciating costs, have augmented the accuracy and efficiency of eDNA surveillance, rendering it an increasingly valuable, explorative method for studying biodiversity in a minimally disruptive and scalable manner. eDNA sampling has been widely used for the surveillance of communicable diseases from wastewater (Xagoraraki and O'Brien 2019; Lee et al. 2022; Roldan‐Hernandez et al. 2025), including SARS‐CoV‐2 (Vicente et al. 2021), poliovirus (Seo et al. 2025) and arboviruses (Lee et al. 2022; Roldan‐Hernandez et al. 2025; Wolfe et al. 2024; Wong et al. 2024; Monteiro et al. 2024), as well as detection of invasive agricultural pests (Farrell et al. 2021) and mosquito vector species (Schneider et al. 2016; Kristan et al. 2023; Odero et al. 2018).

Previously, we conducted a proof‐of‐concept series of controlled laboratory experiments demonstrating that eDNA deposited by 1 s instar larva of *A. stephensi* and *A. aegypti* in 1 L of water was detectable using qPCR and that amplicon‐sequencing of insecticide resistance markers was achievable from eDNA shed by as few as 16–32 s instar larvae in 50 mL of water (Kristan et al. 2023). This study aimed to assess the operational feasibility of eDNA sampling of major malaria vector species in Ghana, by undertaking cross‐sectional sampling of potential aquatic breeding sites and performing a critical evaluation of specimen collection methods and validation of downstream molecular end‐point assays.

## 2 Materials and Methods

### 2.1 eDNA Sampling

**FIGURE 1:**
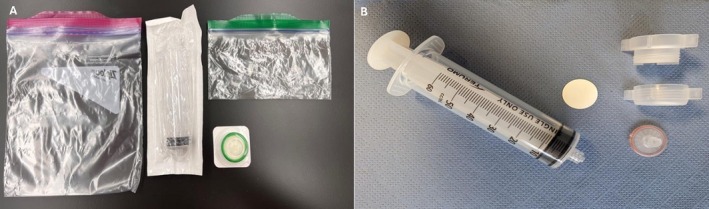
Single‐use (A) or re‐usable (B) 0.22 μm PES syringe filters with 50 mL plastic syringes for eDNA sampling.

To assess the operational feasibility of eDNA detection of mosquito vector species, water samples were collected from potential breeding sites using two types of filtration kits (Figure 1). Each kit contained a 50 mL sterile plastic syringe, a 0.22 μm sterile polyethersulfone (PES) syringe filter (which was either re‐usable or single‐use) and a small zip lock bag labelled with a unique sample ID number.

**FIGURE 2:**
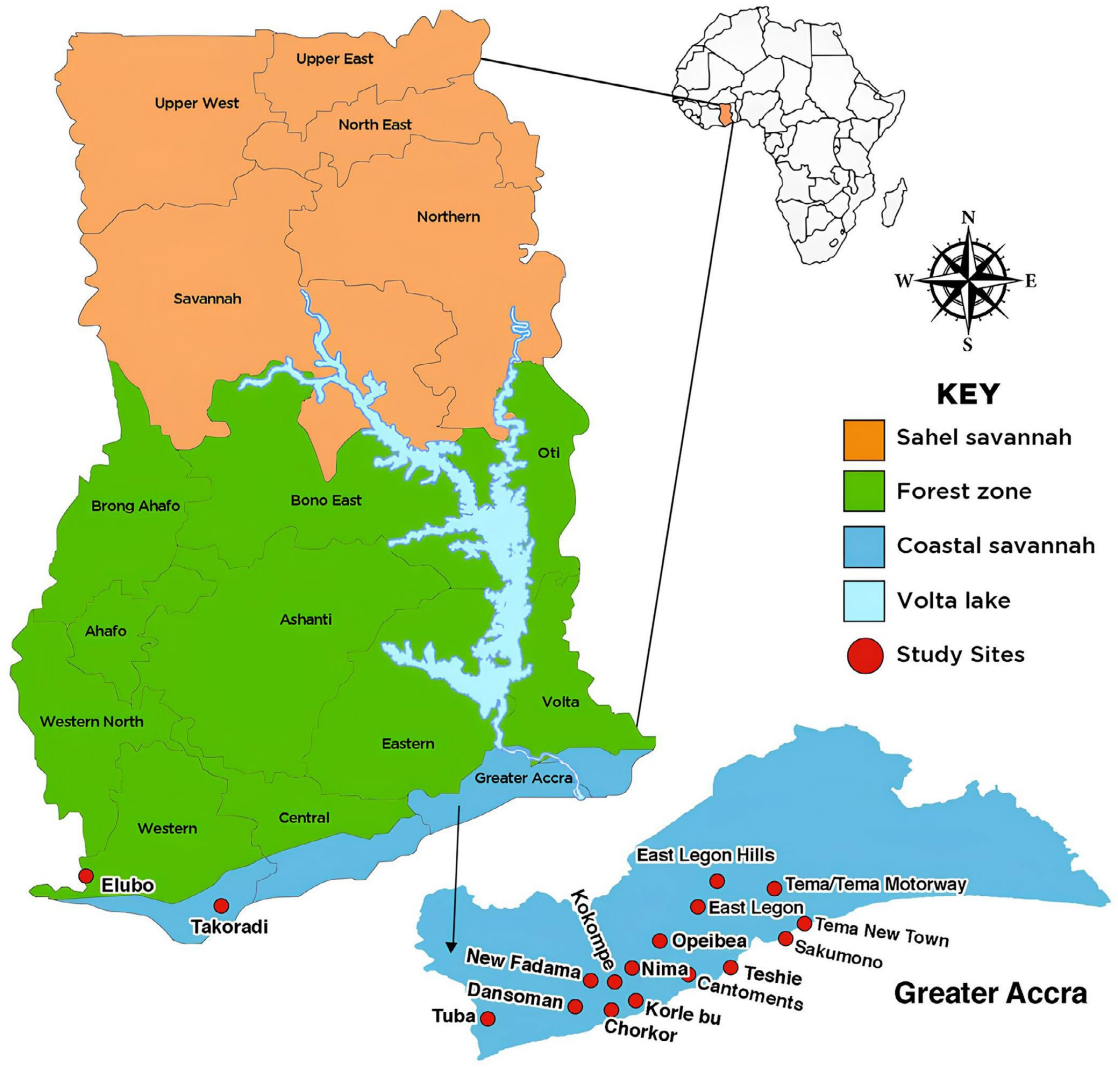
Maps of sampling sites in Greater Accra (Accra) and Western Region (Elubo and Takoradi), Southern Ghana.

**FIGURE 3:**
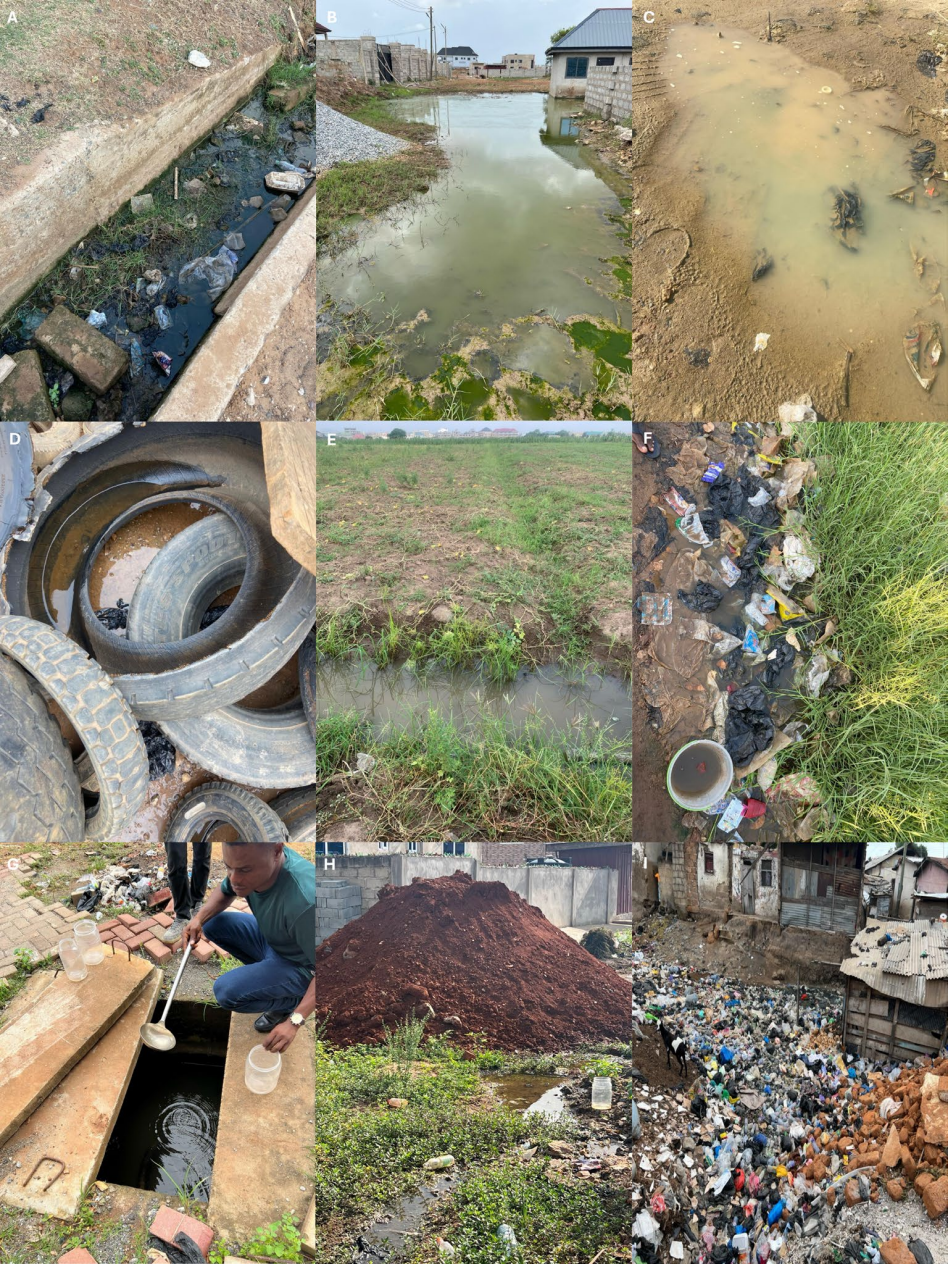
Photos of eDNA sampling locations in Greater Accra and Western Region, Ghana. (A) Upper class neighbourhood ditch in Tema; (B) peri‐urban construction site in Dansoman; (C) peri‐urban puddle in Elubo; (D) peri‐urban tyres in Takoradi; (E) irrigated vegetable farm in Tuba; (F) slum neighbourhood swamp in Tema New Town; (G) upper class neighbourhood concrete water tank in East Legon; (H) middle class neighbourhood puddle from a natural spring in Teshie; and (I) slum neighbourhood ditch in Nima.

**FIGURE 4:**
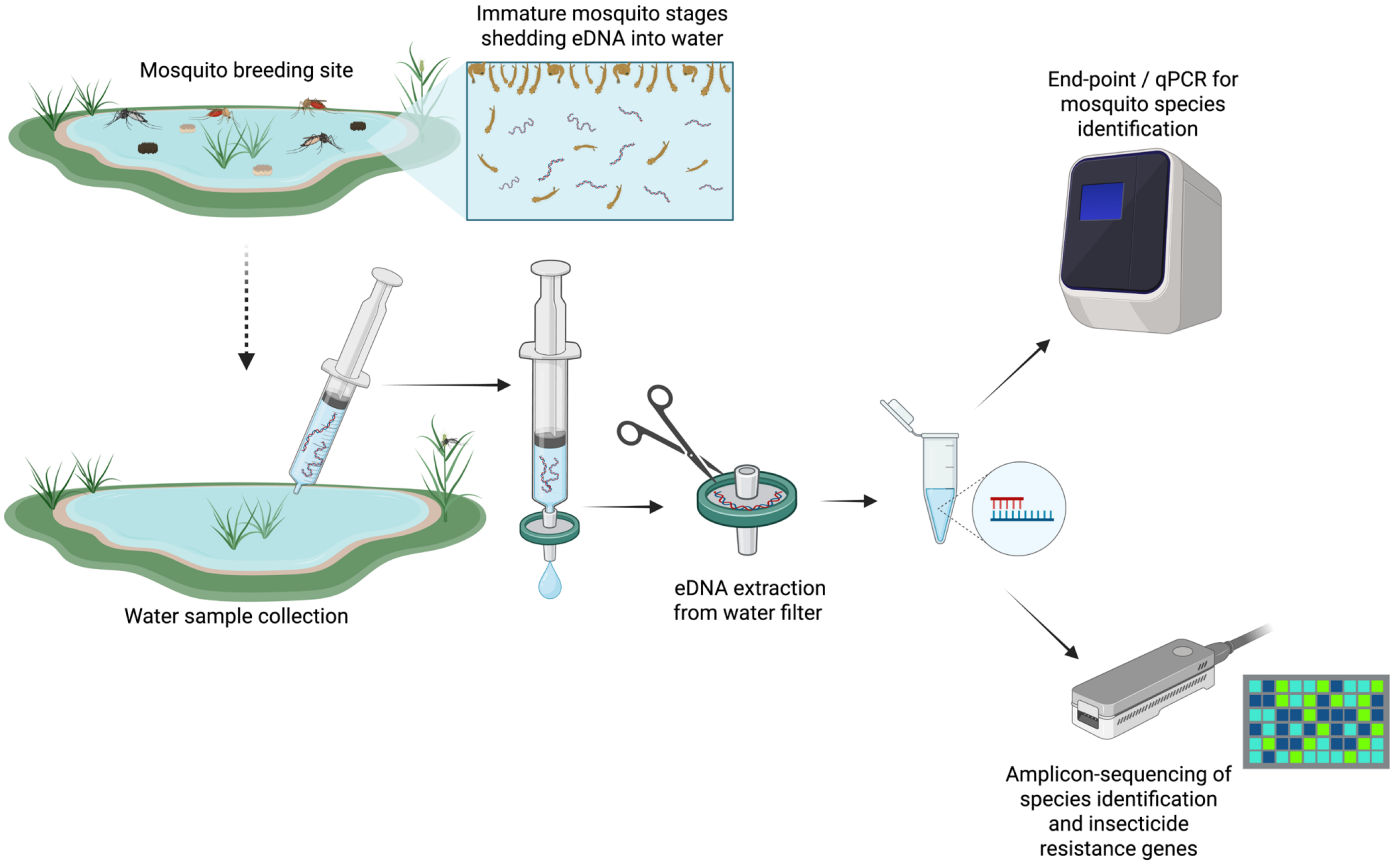
eDNA sampling schematic. Figure created with BioRender.com.

Between 21 November 2023 and 18 January 2024, different breeding site ecologies in Greater Accra and Western Region, Ghana, were systematically searched for stagnant or slow‐flowing water for sample collection, including water tanks, puddles, ditches, tyres, irrigation canals and ponds (Figures 2 and 3). At each aquatic habitat (defined as a single, self‐contained water body), 50 mL of water was collected using a 50 mL sterile plastic syringe (CarePoint Precision, USA), filtered through a 0.22 μm sterile PES syringe filter (single‐use; Membrane Solutions, USA) or re‐usable (Millipore, UK for Millipore Express PES Membrane Filter; Whatman, UK, for Whatman Plastic Filter Holders) (according to the standard operating procedure detailed in Supporting Information Method 1) (Figure 4). In a sub‐set of 36 sites, multiple replicates (two or three) were taken from the same water body to estimate sample concordance. To minimise plastic waste and specimen handling, water samples were collected in the same syringe used for filtration and syringes were used for a single sample and discarded afterwards, including between replicates from the same water body. To avoid contamination in the field, eDNA collectors wore sterile nitrile gloves, which were changed between samples, and filters were immediately sealed in individual zip lock bags and placed in a cooler on ice packs. The re‐usable filter was sterilised overnight after field sampling by disassembling and soaking all plastic components entirely in 10% (v/v) bleach, followed by rinsing in 70% (v/v) ethanol and then boiling deionised water.

Water samples were purposefully collected from breeding sites containing visible mosquito larvae/pupae to directly assess feasibility of vector eDNA detection under natural environmental conditions. eDNA specimens were also collected from six suspected ’unproductive’ sites (with no observable breeding of immature mosquito life stages) in four sites in Greater Accra (Dansoman, the Airport area, Cantoments and Korle‐Bu) and three artificial virgin water bodies constructed at the University of Ghana (UoG) Medical School campus in Korle‐Bu, to assess sampling and assay specificity. Data for each eDNA sample were collected using an electronic survey which recorded the date and time of each sample, global positioning system (GPS) coordinates, elevation (m), ambient temperature (°C), humidity (%), observed visual co‐occupancy and observed presence of immature and/or adult mosquitoes. In addition, water temperature (°C), pH, total dissolved solids (ppm), salinity (ppt) and electrical conductivity (μS/cm) were recorded per site using an environmental probe (SI ProQuatro Multiparameter Meter, Xylem, UK). Additional field observations regarding sampling efficiency, differences in collection methods and challenges were recorded and are reported qualitatively. At the end of each sampling day, eDNA samples were stored at −20°C.

### 2.2 eDNA Extraction and Molecular Detection

eDNA was extracted from filters using the ZymoBIOMICS DNA Microprep Kit (Zymo Research Corporation, USA), with minor modifications to the manufacturer's protocol (Supporting Information Method 2) at UoG. Each filter was added to a ZR BashingBead lysis tube followed by 750 μL ZymoBIOMICS lysis solution and homogenised in a Vortex Genie for 5 min. Next, samples were centrifuged at 8000*g* for 1 min and 700 μL supernatant was transferred to the Zymo‐Spin III‐F filter collection tube, followed by centrifugation at 12,000*g* for 1 min. The Zymo‐Spin III‐F filter was discarded and 1000 μL ZymoBIOMICS DNA binding buffer was added to the filtrate in the collection tube. Next, 800 μL of the mixture was transferred to a Zymo‐Spin IC column in a collection tube and centrifuged at 8000*g* for 1 min; the flow through from the collection tube was discarded and the previous step was repeated. Then, 200 μL ZymoBIOMICS DNA wash buffer 1 was added to the Zymo‐Spin IC column in a new collection tube and centrifuged at 10,000*g* for 1 min; the flow through was discarded. Next, 500 μL ZymoBIOMICS DNA wash buffer 2 was added to the Zymo‐Spin IC column in a collection tube and centrifuged at 8000*g* for 1 min; the flow through was discarded. Then, 200 μL ZymoBIOMICS DNA wash buffer 2 was added to the Zymo‐Spin IC column in a collection tube and centrifuged at 10,000*g* for 1 min. The Zymo‐Spin IC column was transferred to a sterile 1.5 mL microcentrifuge tube and 20 μL ZymoBIOMICS DNase/RNase free water was added directly to the column matrix and the sample was incubated for 1 min, followed by centrifugation at 10,000*g* for 5 min to elute the eDNA. Finally, 600 μL Zymo‐BIOMICS HRC prep solution was added to a new Zymo‐Spin II‐μHRC filter in a new collection tube and centrifuged at 8000*g* for 3 min; the eluted eDNA was transferred to the Zymo‐Spin II‐μHRC filter in a sterile 1.5 mL microcentrifuge and centrifuged at 16,000*g* for 3 min.

Each extraction batch included a negative extraction control, processed in parallel, comprising 50 mL of deionised water filtered through a single‐use 0.22 μm sterile PES syringe filter using a 50 mL sterile plastic syringe. Vector species identification was performed with the two PCR assays most commonly used to identify *Anopheles gambiae* s.l. complex members. End‐point PCR was performed in a final volume of 20 μL containing 10 μM of forward (5′‐TCGCCTTAGACCTTGCGTTA‐3′) and reverse (5′‐CGCTTCAAGAATTCGAGATAC‐3′) primers, 10 μL of HotStart Taq 2X Master Mix (New England Biolabs, UK) and 8 μL of eDNA (Santolamazza et al. 2008). PCR reaction conditions were 10 min denaturation at 94°C, followed by 40 amplification cycles of 94°C for 30 s, 54°C for 30 s and 72°C for 1 min, followed by a final extension at 72°C for 10 min. PCR products (one per eDNA sample) were visualised in 2% agarose gels, stained with ethidium bromide. All PCR runs contained three positive gDNA controls (*A. coluzzii, A. gambiae* s.s. and *A. arabiensis*) and one no‐template control (NTC).

qPCR was performed in a final volume of 10 μL containing 2X PowerUp SYBR Green Master Mix (Applied Biosystems, UK), 10 μM of SINE200Fa (5′‐ATTGCTACCACCAAAATACATGAAA‐3′), SINE200Rd (5′‐GGGGGGGGGAATAATAAGGAACTGCATTTAAT‐3′) and SINE200Re (5′‐GGATGTCTAATAGTCTCAATAGATG‐3′) primers and 4.25 μL of eDNA (Chabi et al. 2019). qPCR reaction conditions were 60 s denaturation at 95°C, followed by 33 amplification cycles of 95°C for 15 s, 60°C for 20 s and 72°C for 10 s, with a final dissociation step of 95°C for 60 s, 55°C for 30 s and a melt ramp up to 95°C with 0.5°C increments. qPCR assays were run in technical triplicate, alongside each positive control and NTCs. Both PCR assays were run at UoG and the London School of Hygiene and Tropical Medicine (LSHTM) in dedicated molecular biology laboratories. All extraction processes and PCR set‐up were performed in laminar flow‐hoods, sterilised with 10% (v/v) bleach and 70% (v/v) ethanol between workflows; laboratories are partitioned into pre‐ and post‐PCR areas.

### 2.3 PCR Data Analysis

Amplified end‐point PCR products of 479 or 249 bp were indicative of *A. coluzzii* or *A. gambiae* s.s., respectively. Amplified qPCR products were 103 or 333 bp for *A. gambiae* s.s. or *A. coluzzii*, respectively. To account for relative differences in qPCR machine temperature calibration, optic sensitivity and software algorithms, we ran genomic DNA, extracted from standard insectary colonies (*A. gambiae* s.s. ZANU, *A. coluzzii* N'Gousso and *A. arabiensis* KGB), as positive controls per assay. At LSHTM, the peak temperature criteria were: > 80°C = *A. coluzzii*, between 80°C and 70°C = *A. gambiae* s.s., < 70°C = *A. arabiensis*. At UoG, the peak temperature criteria were: > 84°C = *A. coluzzii*, between 84°C and 72°C = *A. gambiae* s.s., < 70°C = *A. arabiensis*. An eDNA sample was considered ‘vector negative’ by qPCR if it produced no cycle threshold (*C*_t_) value. An eDNA sample was considered ‘undetermined’ by qPCR if it produced a *C*_t_ value and corresponding *T*_m_ that fell outside the pre‐specified range or produced only a *T*_m_. eDNA sensitivity was measured as the true positive rate, calculated as the proportion of eDNA detected from water bodies with confirmed immature *Anopheles* presence. eDNA specificity was measured as the true negative rate, reflecting the proportion of artificial water bodies lacking immature *Anopheles* breeding, where no eDNA was detected. Differences in eDNA positivity by filter type were assessed using a chi‐square test of proportions. Congruence between eDNA technical replicates was assessed using Cohen's kappa (*κ*) statistic, treating PCR positivity as a binary outcome. Values were interpreted as poor (*κ* ≤ 0), slight (0 < *κ* ≤ 0.2), fair (0.2 < *κ* ≤ 0.4), moderate (0.4 < *κ* ≤ 0.6), substantial (0.6 < *κ* ≤ 0.8) and almost perfect agreement (0.8 < *κ* ≤ 1.0) (Viera and Garrett 2005).

### 2.4 Environmental Data Analysis

We assessed the association between environmental variables and the probability of *Anopheles* vector eDNA detection using mixed‐effects logistic regression. The binary outcome was eDNA detection (presence/absence) per sample. Fixed effects included water temperature, water pH, water depth, total dissolved solids, electrical conductivity, salinity, elevation, ambient temperature and relative humidity. A random intercept was included for breeding site to account for clustering of multiple eDNA samples from the same aquatic site. Model coefficients were exponentiated and reported as odds ratios (OR) with corresponding 95% confidence intervals. Statistical significance was assessed using a two‐sided alpha level of 0.05. All statistical analyses were performed using StataNow/MP 19.5 (StataCorp LLC, College Station, TX).

### 2.5 Nanopore Amplicon‐Sequencing

Previously designed multiplex primers from (Campos et al. 2022; Moss et al. 2024) were used to amplify 13 regions of the *Anopheles* genome, covering key insecticide resistance and species‐specific single nucleotide polymorphisms (SNPs) within nine different genes (Table S3). To enable multiplex amplicon sequencing, forward and reverse primers included 5′ barcodes (8 bp long), with a unique barcode combination assigned to each eDNA sample (Table S4).

Four multiplex reactions and one simplex reaction were used: multiplex 1 (*ITS1, ace1, vgsc‐3, cox1*), multiplex 2 (*ITS2, mtnd4, rdl, vgsc‐2*), multiplex 3 (*vgsc‐4, IGS, gste2*), multiplex 4 (*vgsc‐1, SINE200*) and simplex 5 (*universal cox*). Each PCR reaction had a final reaction volume of 25 μL, containing 0.25 μL Q5 Hot Start High‐Fidelity DNA polymerase (New England Biolabs Ltd., UK), 5 μL of Q5 Hot Start High‐Fidelity 2X Master Mix (New England Biolabs Ltd., UK), 1 μL of eDNA template (2–10 ng), 0.5 μL of 10 mM dNTPs (New England Biolabs Ltd., UK), an average of 0.63 μL of each forward and reverse primer (10 μM) and 15.75 μL of nuclease‐free H_2_O. PCR reaction conditions were: 30 s denaturation at 98°C; followed by 35 cycles of 98°C for 10 s, 57°C for 60 s and 72°C for 60 s; with a final elongation step of 72°C for 2 min.

PCR products were run in 1% agarose gels, stained with SYBR Safe DNA Gel Stain (Invitrogen, UK), to detect presence and size. Barcoded PCR products from different eDNA samples were pooled. Pools were purified using KAPA pure beads (Roche Diagnostics, USA) at a ratio of 0.8:1 (beads: DNA) to remove PCR reagents and excess primers. Purified pools were quantified using the Qubit 4 fluorometer 1X dsDNA HS assay (Invitrogen, UK).

Amplicons were sequenced on the PromethION 2 Solo (P2S) using Native Barcoding Kit 96 V14, with the following protocol (https://nanoporetech.com/document/ligation‐sequencing‐amplicons‐native‐barcoding‐v14‐sqk‐nbd114‐96).

### 2.6 Bioinformatic Analysis

FASTA sequences were base‐called and demultiplexed to separate sequences from individual samples through identification of sample‐specific barcodes. Sequences were then processed to identify SNPs and insertions and deletions (INDELs) using custom python scripts (https://github.com/JosephThorpe/ont_pipeline/ using basecalling model dna_r10.4.1_e8.2_400bps_sup@v5.0.0) and (https://github.com/sophiemoss/anopheles_ampseq/tree/main/nanopore_ampseq_pipeline). Paired‐end reads were mapped to the AgamP4 reference genome (Anopheles_gambiae.AgamP4.dna.toplevel.fa) using minimap2 (version 2.26‐r1175) and variants were called using freebayes (version v1.3.6). SNPs (Table S5) were filtered using bcftools (version 1.17) for QUAL > 30 and FMT/DP > 10 and annotated using bcftools csq. Annotated SNPs were further filtered for ALT allele depth ≥ 20 and frequencies were calculated. Amplicons with 20× read depth coverage were excluded from frequency calculations. Species specific amplicons IGS, SINE200, ITS1, ITS2 and Mt_ND4 were extracted and consensus fasta files were created for each sample. Blastn was then used to query these consensus fasta files against a local nucleotide database with parameters ‐evalue 1e‐10 ‐max_target_seqs 5 ‐max_hsps 1. Species results were visualised using matplotlib.

## 3 Results

### 3.1 eDNA Sensitivity, Specificity and Environmental Factors

One hundred and eighty‐three eDNA samples (55 re‐usable filters and 128 fixed, single‐use filters) were collected from 116 breeding habitats across 19 sites in Greater Accra Region, of which immature *Anopheles* spp. were observed breeding in 93.44% (171/183). Most water samples were collected in peri‐urban areas (47.54%; 87/183), upper‐class neighbourhoods (16.94%; 31/183), middle class neighbourhoods (14.21%; 26/183) and irrigated rice farms (12.02%; 22/183). The majority of water sources were puddles (37.70%; 69/183), ditches (24.04%; 44/183) and water tanks (8.74%; 16/183). PCR data were generated for 91.23% (156/171) of eDNA samples with observable vector breeding, of which *Anopheles* eDNA was detected in 76.28% (119/156) of samples. In breeding sites with no observable immature *Anopheles* spp., eDNA was detected in 63.64% (7/11) of samples. Among all eDNA positive breeding sites, the predominant vector species was *A. coluzzii* (56.35%; 71/126), with comparable proportions of *A. gambiae* s.s. (23.81%; 30/126) and co‐habitation of the two species (19.84%; 25/126). Positivity rates did not differ significantly by filter type (65.45%; 36/55 and 70.31%; 90/128 for re‐usable and fixed, single‐use filters, respectively; *χ* ^2^ = 0.423, *p* = 0.515). In ‘artificial’ vector negative water bodies, eDNA specificity was 100%, with no false positive results. In ‘unproductive’ water bodies in the community, eDNA was detected in 66.67% (4/6) of samples. Agreement between eDNA technical replicates per aquatic habitat was ‘fair’ (*κ* = 0.213, *p* = 0.039). qPCR *C*_t_ values ranged from 10.61 to 39.94, with a median of 31.34.

No environmental variables were associated with eDNA detection after adjustment for multiple samples collected per breeding site (Table 1). Similarly, there was no association between breeding site type and eDNA detection (OR: 1.78 [95% CI: 0.53–5.99]; *p* = 0.354).

**TABLE 1:** Summary of eDNA study site environmental characteristics.

Environmental variable	Mean	Standard deviation	Range	Odds ratio [95% CI]^a^	*p*
Water temperature (°C)	30.72	2.67	25.8–35.8	0.88 [0.65–1.21]	0.447
Water pH	8.38	0.54	7.48–10.06	2.26 [0.49–10.42]	0.296
Water volume (cm^3^)	87.40	168.38	2.00–1080.00	1.02 [0.99–1.04]	0.181
Total dissolved solids (ppm)	776.98	1555.58	1.02–6680.00	1.00 [0.99–1.00]	0.932
Electrical conductivity (μS/cm)	1446.53	2766.60	2.37–12128.00	1.00 [0.99–1.00]	0.822
Salinity (ppt)	1.09	1.39	0.04–5.7	0.89 [0.40–1.97]	0.768
Elevation (m)	26.86	18.52	0.00–85.00	0.99 [0.88–1.12]	0.931
Ambient temperature (°C)	29.02	1.65	26.00–33.00	0.87 [0.42–1.80]	0.702
Ambient humidity (%)	54.67	21.41	29.00–86.00	1.00 [0.96–1.05]	0.839

^a^ Odds ratio reported from mixed effects logistic regression to identify environmental predictors of eDNA detection, with fixed effects included for environmental variables and a random intercept included for breeding site to account for multiple eDNA samples per breeding site.

### 3.2 eDNA Detection of Insecticide Resistance Mutations

**FIGURE 5:**
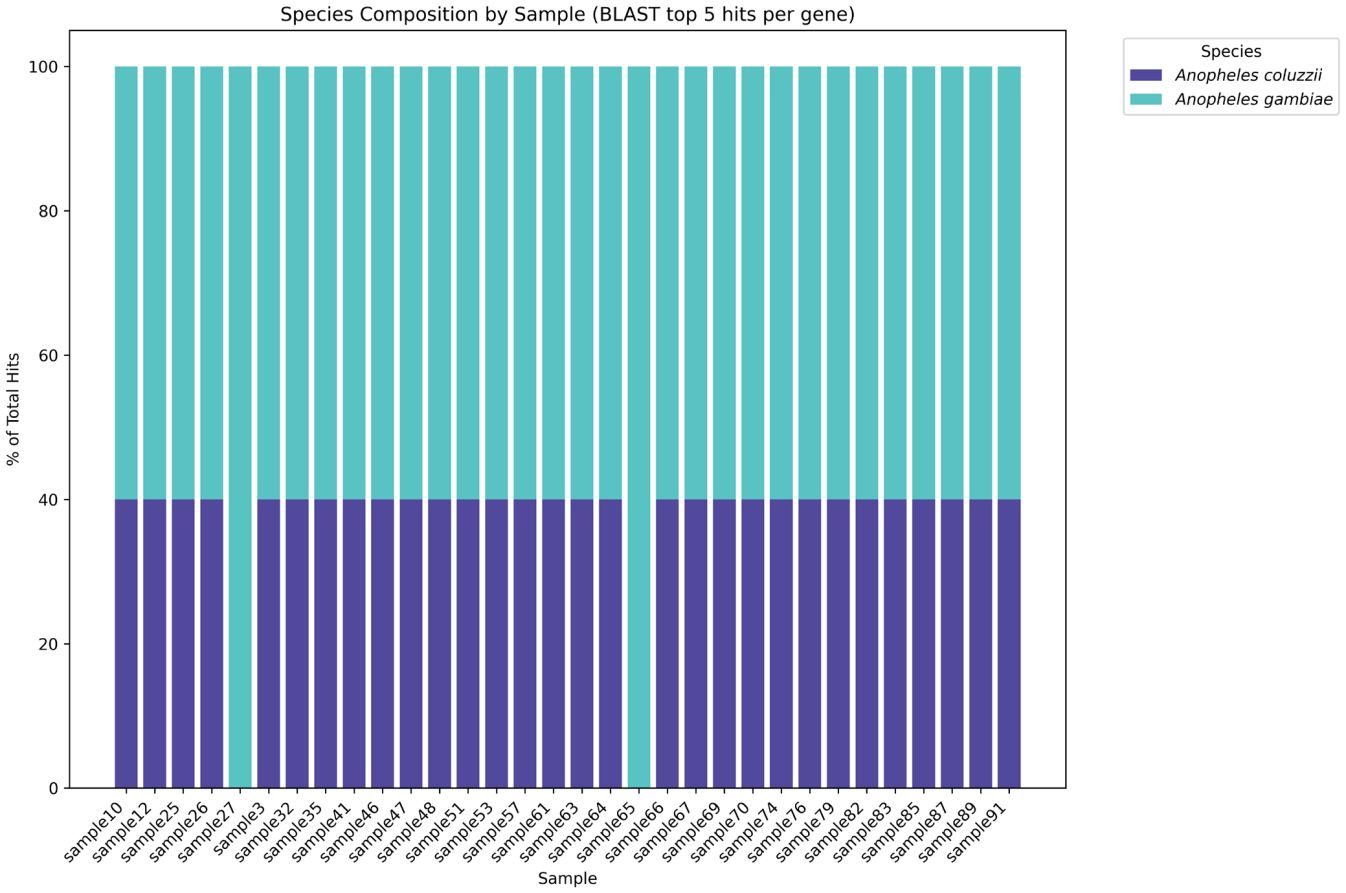
Species composition of each eDNA sample (Blastn top 5 hits per amplicon).

Amplicons were successfully generated and sequenced from 32 eDNA samples, which had previously yielded positive qPCR results (Table S1). The proportion of amplicons generated at ≥ 10× coverage per eDNA sample was variable, ranging from 56.3% of samples amplifying VGSC1_D4, to all samples amplifying COX1, IGS, ITS1, ITS2, MtND4, SINE200 and VGSC1_D2 (Table S2). Similarly, the proportion of eDNA samples with ≥ 10× coverage per amplicon was variable, ranging from 53.8% to 100% (Table S1). Eight known insecticide resistance mutations were detected in eDNA samples (Table 2). The highest mutation frequencies were observed for *rdl‐*A296G (0.554), *vgsc‐*L995F (0.500) and *vgsc‐*A1746S (0.214). *gste2‐*L120F was identified for the first time in Ghana at a frequency of 6.3% in eDNA from shared *A. coluzzii*—*A. gambiae* s.s. breeding sites. Five taxonomic markers (COX1, IGS, ITS1, ITS2 and MtND4) were used to assign sequencing reads to vector species. Thirty samples which underwent amplicon‐sequencing were classified as a mix of eDNA shed by *A. coluzzii* and *A. gambiae* s.s. (Figure 5), consistent with PCR identification results. Among taxonomic markers, only IGS, ITS1 and ITS2 were able to distinguish between *A. gambiae* s.l. complex members (Figure S1). All genotyping data are available in Supporting Information Data S1.

**TABLE 2:** Summary of known insecticide resistance mutations present in eDNA samples.

CHR	POS	REF	ALT	Amplicon	ALT alleles	# Samples with coverage	FREQ	Known SNP
2 L	2,422,652	A	T	VGSC1_D2	32	32	0.500	L995F
2 L	2,429,617	T	C	VGSC1_D3	1	26	0.019	I1527T
2 L	2,429,623	T	G	VGSC1_D3	1	26	0.019	F1529C
2 L	2,430,424	G	T	VGSC1_D4	3	7	0.214	A1746S
2 L	25,429,236	C	G	RDL	31	28	0.554	A296G
2R	3,492,074	G	A	ACE1	5	16	0.156	G280S
3R	28,598,057	G	T	GSTE2	3	24	0.063	F120L
3R	28,598,062	G	C	GSTE2	5	24	0.104	L119V

Abbreviations: ACE1, acetylcholinesterase; ALT, alternate; CHR, chromosome; FREQ, frequency; GSTE2, glutathione‐*S*‐transferase epsilon 2; POS, position; RDL, resistance to dieldrin; REF, reference; SNP, single nucleotide polymorphism; VGSC, voltage‐gated sodium channel.

## 4 Discussion

By comparison to conservation and biodiversity initiatives, eDNA surveillance in medical and veterinary parasitology/entomology has been more limited, with the majority of studies focusing on parasitic trematodes and their intermediate hosts and mosquito vectors to a lesser extent (Thomsen and Willerslev 2015; Schneider et al. 2016; Odero et al. 2018; Sengupta et al. 2022; Wittwer et al. 2024; Boerlijst et al. 2019; Sakata et al. 2022; Krol et al. 2019; Soler et al. 2025). To improve our capacity in the latter regard and our ability to respond to real‐time emergent VBD threats, this study undertook cross‐sectional sampling of malaria vector breeding sites in Ghana, to assess the operational feasibility of eDNA detection, optimise specimen collection and critically evaluate available downstream molecular assays. eDNA deposited by wild, immature *A. gambiae* s.l., isolated from a 50 mL water sample, was detectable using both end‐point PCR and qPCR, achieving high levels of sensitivity (76.28%) and specificity (100%). Detection levels reported herein are consistent with previous ecological studies, which have conducted eDNA monitoring of different biological taxa (Sieber et al. 2020; Shuai et al. 2025; Rees, Bishop, et al. 2014). Furthermore, we were able to recover PCR amplicons covering eight known insecticide resistance mutations from eDNA collected in natural breeding sites. To our knowledge, this study represents the first proof‐of‐concept demonstrating that eDNA can be used for simultaneous identification of *Anopheles* vector species and circulating insecticide resistance alleles directly from aquatic habitats in the community, including the first report of the mutation *gste2*‐F120L in Ghana. Study findings also validate the potential to couple new portable sequencing technologies with eDNA monitoring to provide accurate population‐level estimates of insecticide resistance mutations and vector species distribution.

We evaluated the field usability of two commonly available PES filter systems (re‐usable or fixed, single‐use filters), serving distinct purposes. The fixed, single‐use PES filter required no assembly, limited expertise to use and allowed for easy sample transportation without contamination concerns. The relative disadvantages of this system are that these filters are more challenging to open to retrieve the membrane for nucleic acid extraction, they generate more plastic waste and are a little slower and slightly harder to filter water through, compared to re‐usable filters. The re‐usable filters generated minimal plastic waste, allowed the filter pore size to be changed (e.g., to increase pore size to 0.45 or 0.7 μm) and were marginally easier and quicker to use for filtration (approximately 30 s versus 1 min between filter types). However, re‐usable filters are dependent on expertise and dexterity to assemble, required sterilisation between collections, limiting the number of samples collected per day, and some of the smaller parts were easily lost during field work. In general, volumes of water filtered for eDNA sampling are greater (> 1 L) than used in this study and filtered using vacuum pumps, which require electrical power; with sampling strategies determined by water type and turbidity; larger volumes are collected from marine environments where eDNA is presumed to be more dilute, and smaller volumes sampled from sediment‐rich environments, such as waste water (Bylemans et al. 2018; Deiner et al. 2017; Goldberg et al. 2016; Sengupta et al. 2019).

One of the main strengths of eDNA‐based surveillance is its methodological flexibility, allowing aspects of sampling and analysis to be tailored to specific hypotheses and vector/pathogen species of interest (Sengupta et al. 2022; Wittwer et al. 2024; Deiner et al. 2017; Goldberg et al. 2016; Allen et al. 2024; Bohmann et al. 2014; Ruppert et al. 2019; Amarasiri et al. 2021). Our eDNA sampling strategy was intentionally designed with citizen scientists as the primary end‐users, ensuring accessibility, ease and minimal requirements for training or technical infrastructure. While optimised for grassroots implementation, this framework is readily adaptable by academic researchers or public health programmes. For example, increasing sample volume can improve eDNA yield and detection probability, which will be more critical when monitoring emergent, invasive species/pathogens circulating at low abundance in the environment. Furthermore, once eDNA collection and downstream processing capacity is established, greater numbers of samples can be analysed faster and cheaper than ‘gold standard’ larval dipping, which becomes labour intensive when performing larval morphological identification and/or rearing larvae to adulthood to confirm species. Traditional larval surveys are still important for precision estimates of immature vector densities; however, eDNA has the potential to be utilised in an integrated approach. eDNA can be used for rapid surveillance with broader spatial and temporal coverage, especially when engaging citizen scientists, facilitating targeted follow‐up of positive/suspected breeding sites for additional vector larval or adult trapping (Biggs et al. 2015; Bonicalza et al. 2024; Leerhoi et al. 2024; Couton et al. 2023; Lin et al. 2021).

We encountered several operational challenges during sample collection and analysis, which require future consideration during the design and execution of eDNA surveillance. When sampling water bodies containing significant levels of organic material, we often struggled to filter a full 50 mL of water, due to filter clogging, therefore, eDNA sensitivity may have been underestimated in some samples. There is increasing evidence of ecological plasticity of *A. gambiae* s.l., particularly, *A. coluzzii*, to colonise breeding sites rich in organic detritus and man‐made containers, allowing vectors to now infiltrate peri‐urban and urban environments (Kudom et al. 2012; Longo‐Pendy et al. 2021). Filter pore size could be increased to facilitate easier sample collection and/or water samples containing larger debris could be pre‐filtered through a large pore filter first (e.g., 5 μm). We chose to trial the smallest available pore size to maximise detection sensitivity and enable the collection of microbial eDNA for future microbiome association studies, given the increasing evidence for a role of certain endosymbionts in vector insecticide resistance phenotype (Pelloquin et al. 2021; Omoke et al. 2021; Dada et al. 2018). We also observed multiple qPCR samples report no *C*_t_ value accompanied by a *T*_m_ peak or *C*_t_ values accompanied by an inaccurate *T*_m_; both types of samples were excluded from analysis. These observations may reflect very low/undetectable target eDNA, detection of primer dimers, degraded eDNA and/or presence of environmental inhibitors (e.g., excess salts or organic solvents); the latter were not directly investigated, which is a limitation of this study. eDNA is highly fragmented due to exposure to UV, microbial activity, oxidation, temperature fluctuations and chemical degradation, typically ranging from 50 to 300 base pairs (Kirtane et al. 2021; Riaz et al. 2011; Collins et al. 2018; Rees, Maddison, et al. 2014; Saito and Doi 2021; Jo et al. 2017), whereas our qPCR assay targeted amplicons of 103 and 333 bp, the latter of which may have been at the upper limit of eDNA fragment length in more degraded specimens. To circumvent issues of non‐specificity and to improve detection levels, a TaqMan‐based probe assay could be designed for *A. gambiae* s.l. species or droplet digital PCR (ddPCR) used instead; the latter platform is less susceptible to environmental inhibition and offers higher sensitivity, especially at low DNA concentrations (< 1 copy/μL reaction) (Guri et al. 2024; Mauvisseau, Davy‐Bowker, et al. 2019). Finally, our inter‐replicate concordance was slightly lower than anticipated, albeit comparable with other published studies (Parsley et al. 2024; Beentjes et al. 2019; Mauvisseau, Burian, et al. 2019), which we attribute to low eDNA concentrations, heterogeneity in eDNA distribution in natural water bodies and stochastic amplification effects during PCR. We also recommend the collection of multiple eDNA replicates per aquatic site to maximise detection levels, particularly, of low abundance taxa (de Souza et al. 2016; Buxton et al. 2023).

Despite this being a pragmatic technology development study, results yielded several other relevant entomological observations. We were able to isolate eDNA from the two major malaria vector species in southern Ghana (*A. gambiae* s.s. and *A. coluzzii*) (Dortey et al. 2025; Akuoko et al. 2024; Sabtiu et al. 2025) in aquatic bodies with no apparent immature stages or resting adult mosquitoes at the time of sampling. This demonstrates the potential for eDNA monitoring to identify cryptic vector breeding sites, detecting evidence of recent or transient vector activity, in areas which may not be under routine entomological surveillance. We also observed substantial *A. gambiae* s.l. breeding in peri‐urban and urban areas, co‐habitation of *A. gambiae* s.s. and *A. coluzzii* and shared breeding sites with *Culex* and *Aedes* spp. The traditional paradigm of *A. gambiae* s.l. complex breeding site preference indicates that *A. gambiae* s.s. prefer temporary, clean, unpolluted, freshwater habitats, while *A. coluzzii* thrives in permanent or semi‐permanent, shaded environments, with tolerance to polluted or organically enriched waters, contributing to their ecological segregation (Longo‐Pendy et al. 2021; Ebhodaghe et al. 2024; Mattah et al. 2017). Further investigation is required to determine whether members of the *A. gambiae* s.l. complex are adapting to urbanised settings in Ghana and the consequences this may have on exacerbating local, urban malaria transmission (Savi et al. 2024, 2021).

## 5 Conclusions

This study validated the use of eDNA for simultaneous identification of *Anopheles* vectors and circulating insecticide resistance alleles directly from aquatic habitats in the community.

eDNA deposited by wild, immature *A. gambiae* s.l., isolated from a 50 mL water sample, was detectable using both end‐point PCR and qPCR, achieving high levels of sensitivity (76.28%) and specificity (100%). Furthermore, we recovered PCR amplicons covering eight known insecticide resistance mutations from eDNA collected in natural breeding sites, including the first report of the mutation *gste2*‐F120L in Ghana. Our eDNA sampling methodology was designed for citizen scientists as end‐point users; we recommend fixed, single‐use filters for sampling 50 mL of water as a highly feasible, sensitive and specific community‐led mosquito vector surveillance tool, which requires minimal training or technical infrastructure.

## Author Contributions

M.K., Y.A.A. and L.A.M. designed the study. Y.A.A., M.K., L.A.M., Y.B., A.R.M.S., I.K.S. and B.C.O. performed the eDNA sampling. M.K., J.D.A., A.A. and L.A.M. performed the PCR analysis. S.M., T.G.C. and S.C. conducted the amplicon sequencing and bioinformatics analysis. K.L.F.C. supported data analysis and visualisation. S.C. and J.L. provided project oversight and funding. L.A.M., M.K., S.M. and Y.A.A. drafted the manuscript, which was revised by co‐authors. All authors read and approved the final manuscript.

## Funding

This work was supported by the Foreign, Commonwealth and Development Office (PO8615), National Institutes of Health (AI186018 and TW011513), Biotechnology and Biological Sciences Research Council (BB/X018156/1), Medical Research Council (MR/X005895/1) and Engineering and Physical Sciences Research Council (EP/Y018842/1).

## Conflicts of Interest

The authors declare no conflicts of interest.

## Supporting information


**Data S1:** All quality filtered SNPs are included in the supplementary excel file (Supplementary_Data_combined_genotyped_filtered_formatted.snps.trans).

**Table S1:** Proportion of samples with ≥ 10× sequencing coverage per amplicon.
**Table S2:** Proportion of amplicons with ≥ 10× sequencing coverage per sample.
**Figure S1:** Vector species composition per informative taxonomic amplicon.
**Supplementary Method 1:** Standard operating procedure for eDNA sampling.
**Supplementary Method 2:** Standard operating procedure for eDNA extraction.
**Table S3:** Target amplicons covering key insecticide resistance and species‐specific SNPs (AgamP4 reference genome).
**Table S4:** Primers for insecticide resistance amplicon sequencing for the *Anopheles gambiae* s.l. complex.
**Table S5:** Target SNPs, which have been associated with insecticide resistance in the *Culicidae* family. Genomic positions according to the AgamP4 reference genome.

## Data Availability

Sequence data generated by this study will be available in the European Nucleotide Archive (https://www.ebi.ac.uk/ena/browser/home) BioProject PRJEB89240 (accession numbers: ERR15107760 and ERR15107791). All quality filtered SNPs are included in Data S1.
