# Supplementary material for: Operationalising Environmental DNA (eDNA) Detection of Major Malaria Vector Species in Ghana
Source: Mol Ecol Resour. 2026 Jul 28;26(5):e70154. doi: 10.1111/1755-0998.70154 (PMC13409677; doi:10.1111/1755-0998.70154)
Supplement: Supplementary file 2 — Table S1: Proportion of samples with ≥ 10× sequencing coverage per amplicon. Table S2: Proportion of amplicons with ≥ 10× sequencing coverage per sample. Figure S1: Vector species composition per informative taxonomic amplicon. Supplementary Method 1: Standard operating procedure for eDNA sampling. Supplementary Method 2: Standard operating procedure for eDNA extraction. Table S3: Target amplicons covering key insecticide resistance and species‐specific SNPs (AgamP4 reference genome). Table S4: Primers for insecticide resistance amplicon sequencing for the Anopheles gambiae s.l. complex. Table S5: Target SNPs, which have been associated with insecticide resistance in the Culicidae family. Genomic positions according to the AgamP4 reference genome. [file MEN-26-e70154-s002.docx]

**SUPPLEMENTARY MATERIAL**

**Supplementary Table 1.** Proportion of samples with ≥10x sequencing coverage per amplicon.

| **Sample** | **Success (% of amplicons with coverage ≥10x)** | **qPCR Ct value** |
| --- | --- | --- |
| sample10 | 76.9 | 36.24 |
| sample12 | 100 | 37.74 |
| sample25 | 92.3 | 35.95 |
| sample26 | 100 | 35.59 |
| sample27 | 100 | 29.39 |
| sample3 | 76.9 | 35.46 |
| sample32 | 100 | 38.02 |
| sample35 | 92.3 | 34.56 |
| sample41 | 100 | 35.26 |
| sample46 | 92.3 | 34.23 |
| sample47 | 100 | 32.85 |
| sample48 | 84.6 | 36.55 |
| sample51 | 100 | 35.63 |
| sample53 | 92.3 | No Ct |
| sample57 | 69.2 | 37.68 |
| sample61 | 100 | 36.74 |
| sample63 | 100 | 29.18 |
| sample64 | 100 | 28.43 |
| sample65 | 100 | 26.67 |
| sample66 | 100 | 32.42 |
| sample67 | 69.2 | 38.54 |
| sample69 | 92.3 | 36.08 |
| sample70 | 92.3 | 37.86 |
| sample74 | 100 | 38.79 |
| sample76 | 100 | 37.56 |
| sample79 | 53.8 | 37.74 |
| sample82 | 100 | 35.55 |
| sample83 | 92.3 | 36.24 |
| sample85 | 100 | 33.25 |
| sample87 | 76.9 | 35.96 |
| sample89 | 92.3 | 37.72 |
| sample91 | 100 | 35.95 |

**Supplementary Table 2.** Proportion of amplicons with ≥10x sequencing coverage per sample.

| **Amplicon** | **Success (% of samples with coverage ≥10x)** |
| --- | --- |
| ACE1 | 78.1 |
| COX1 | 100 |
| GSTE2 | 90.6 |
| IGS | 100 |
| ITS1 | 100 |
| ITS2 | 100 |
| MtND4 | 100 |
| RDL | 96.9 |
| SINE200 | 100 |
| VGSC1_D1 | 78.1 |
| VGSC1_D2 | 100 |
| VGSC1_D3 | 96.9 |
| VGSC1_D4 | 56.3 |

ACE1: acetylcholinesterase; COXI: cytochrome c oxidase subunit I; GSTE2: glutathione-*S*-transferase epsilon 2; IGS: intergenic space region; ITS: internal transcribed spacer; MtND4: mitochondrial nicotinamide adenine nucleotide dehydrogenase subunit 4; RDL: resistance to dieldrin; SINE200: Short INterspersed Elements; VGSC: voltage-gated sodium channel

**Supplementary Figure 1.** Vector species composition per informative taxonomic amplicon.


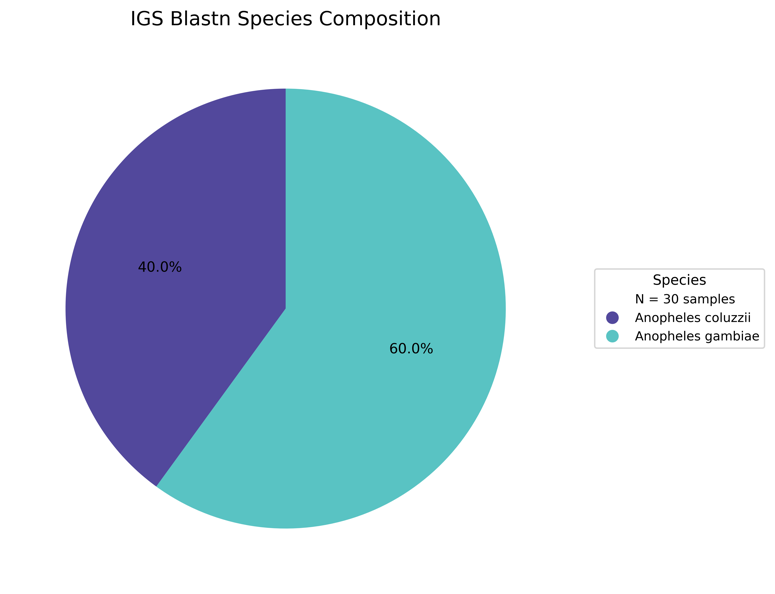

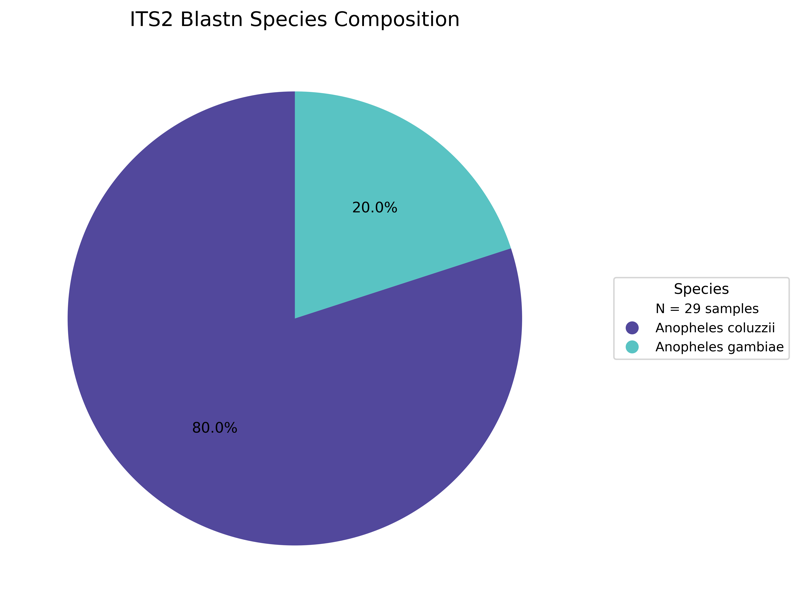

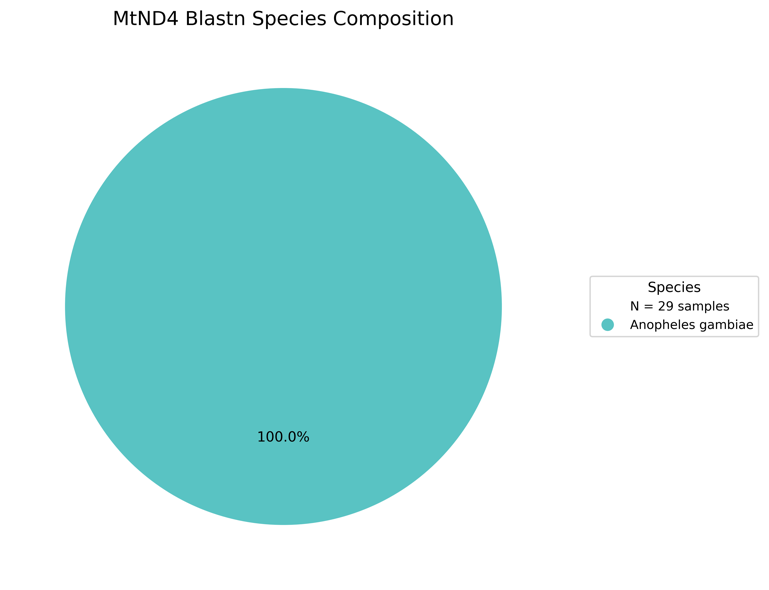

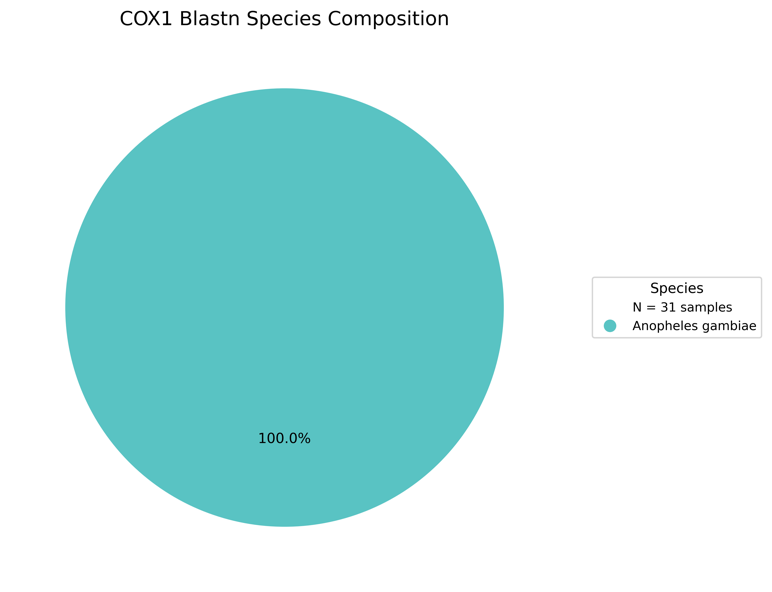

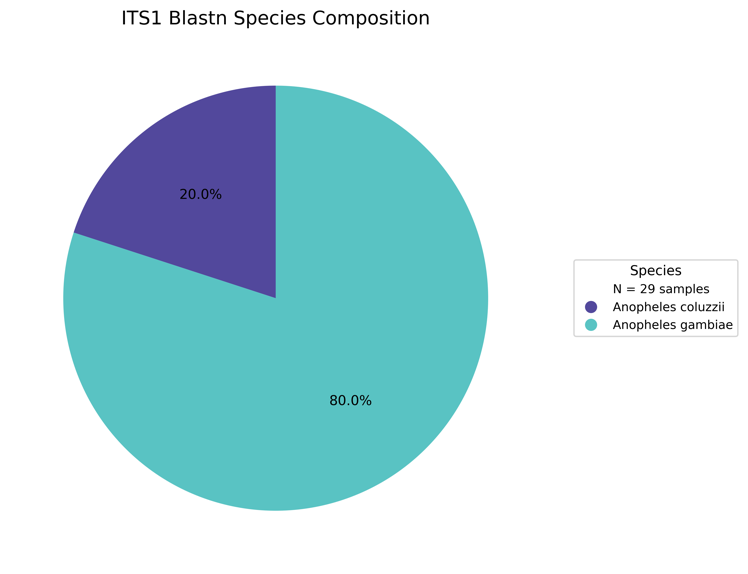


**Supplementary Method 1.** Standard Operating Procedure for eDNA sampling.

Water samples can be collected using one of the two different types of filters:

1.
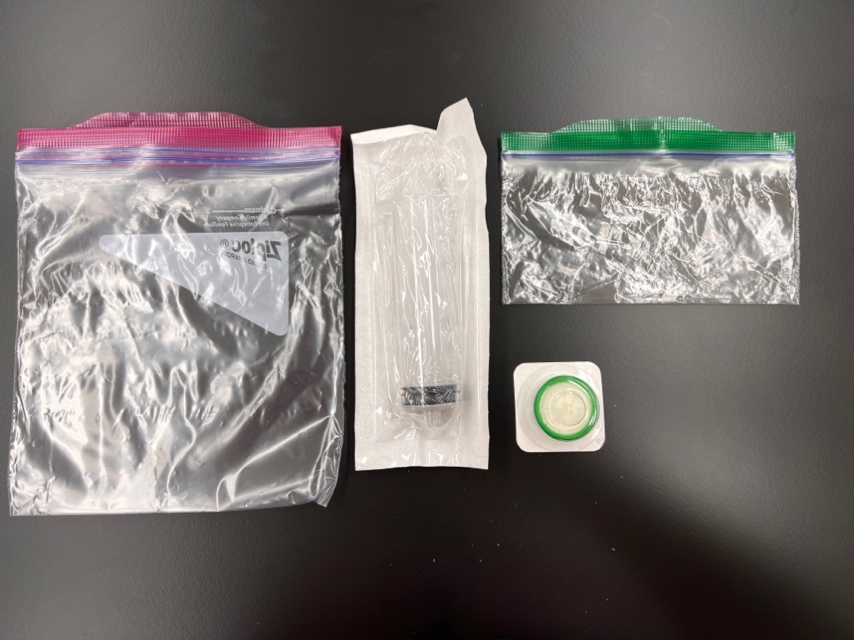
**Fixed filters** (PES membrane, 0.22 µm pore size)

To take samples in one location, you will need:

- one 50ml single-use luer-lock plastic syringe
- three 0.22µm water filters
- one ziplock bag

1. **Reusable filters** (MCE membrane, 0.22 µm pore size)

The case of these can be re-used after thorough sterilization between uses to avoid contamination, while filter papers need to be bought separately.


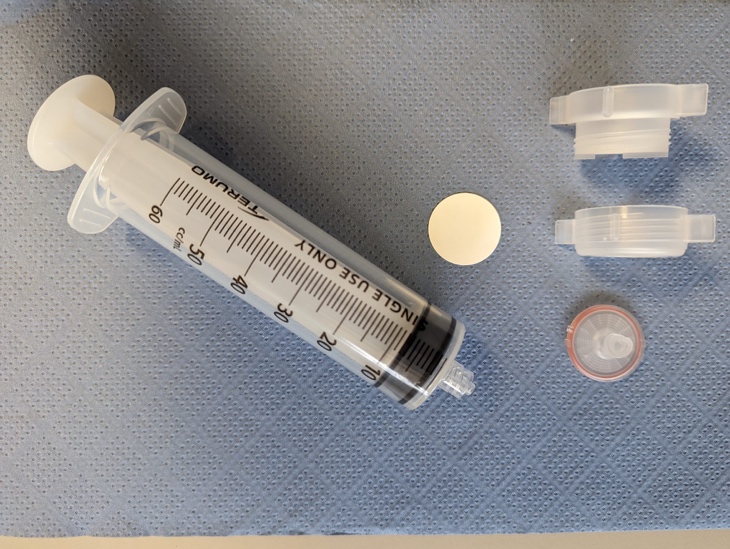


To take samples in one location, you will need:

- one 50ml single-use luer-lock plastic syringe
- three pre-assembled 0.22µm water filters, each packed in a separate ziplock bag

Assembly of re-usable filters:

Assemble all the parts as shown in the diagram below. The cap with the o-ring has notches in it that have to fit into the notches in the rim of the base. If fitted correctly, the winged bits of assembly ring and base should be parallel once the filter is assembled. When assembling the filter make sure the filter membrane does not get contaminated. If possible, pre-assemble them in the laboratory, wearing gloves and using sterile forceps while assembling. Pack each assembled filter into a ziplock bag.


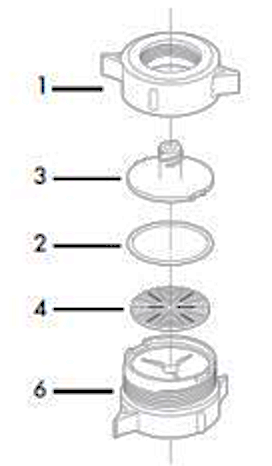


Assembly ring

Cap with o-ring

Filter membrane

Support grid

Base

Water sample collection if filters are available:

1. Three replicates of ~50ml of water will be removed from the source and filtered through a fixed 0.22μm sterile syringe filter.

OR

1. Three replicates of 50ml of water will be removed and filtered through a reusable 0.22μm sterile syringe filter.

Note: the same syringes can only be used for the samples taken in the same locality.

1. Remove the syringe from the wrapper and collect the water sample without the filter attached. Try to collect water without clumps of algae or other large solid particles as they can block the syringe and/or filter.
2. Once the sample is in the syringe, open and attach the fixed or re-usable filter to the syringe by screwing it on clockwise. Push the water through the filter until the syringe is completely empty.


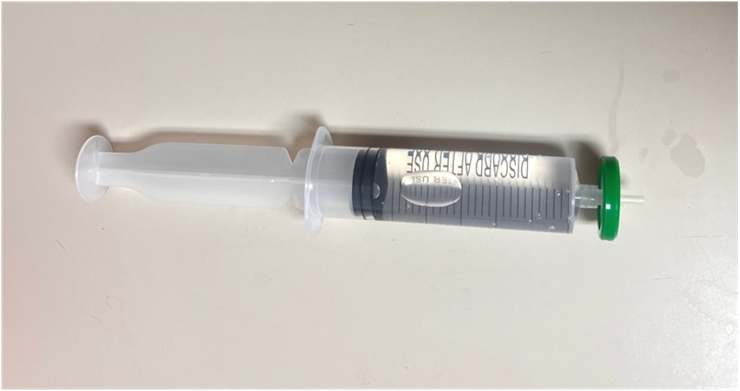

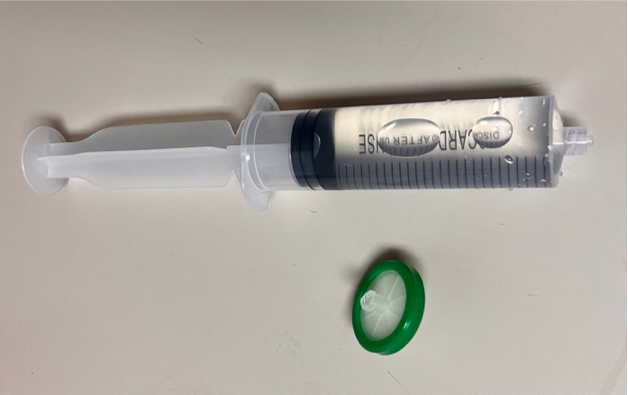


Note: when you start pushing water through the filter, do not use a lot of force, so the water keeps flowing through the filter at a steady pace. It might get very difficult towards the end but try to filter as much water as possible. If you notice the filter/syringe is clogged, remove the filter and try to remove the blockage if possible (with a toothpick or a twig). Then screw the filter back on and continue. If you only manage to filter a small amount of water (10-20ml) keep the filter but make a note of this in the form.

1. Remove the filter from the syringe. When using a fixed filter, place all three replicates from one site into the same small ziplock bag. When using re-usable filters, place each into its own ziplock bag after use.


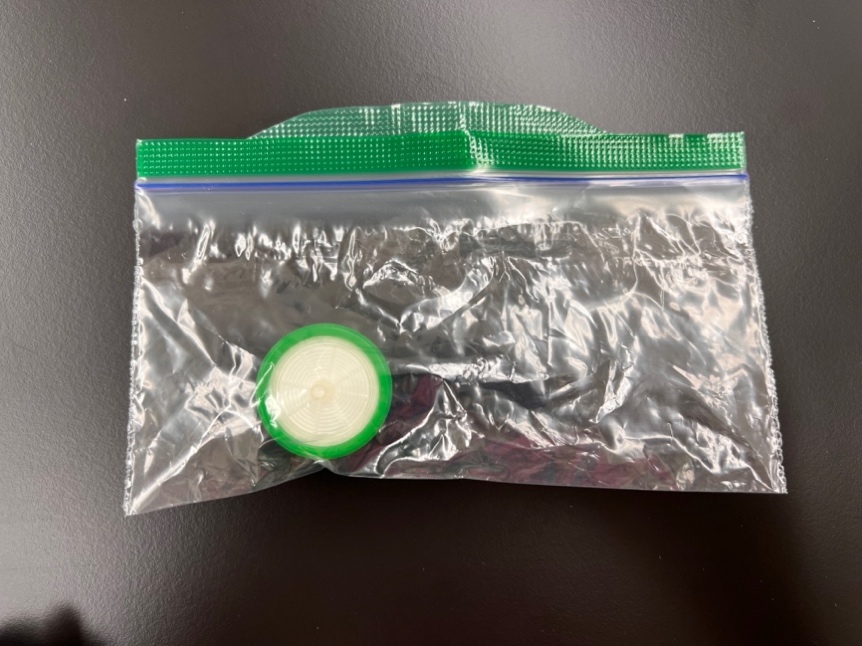


1. Label each ziplock bag with used filter with: name of location (e.g. Nima #2, Teshie #1, etc.), date of collection, number of sample (e.g. eDNA 6).

Note: you can start the numbering of samples sequentially (from 1) each day on ziplock bags and enter the corresponding sample code into the data collection form. However, samples should be re-numbered according to the sample number in the database once stored for freezing.

1. Store at 4^o^C (in fridge or cool box) if possible or at room temperature until you return to the lab.
2. Place the syringe back into its wrapper and collect any other disposable wrappers, to discard whenever possible.


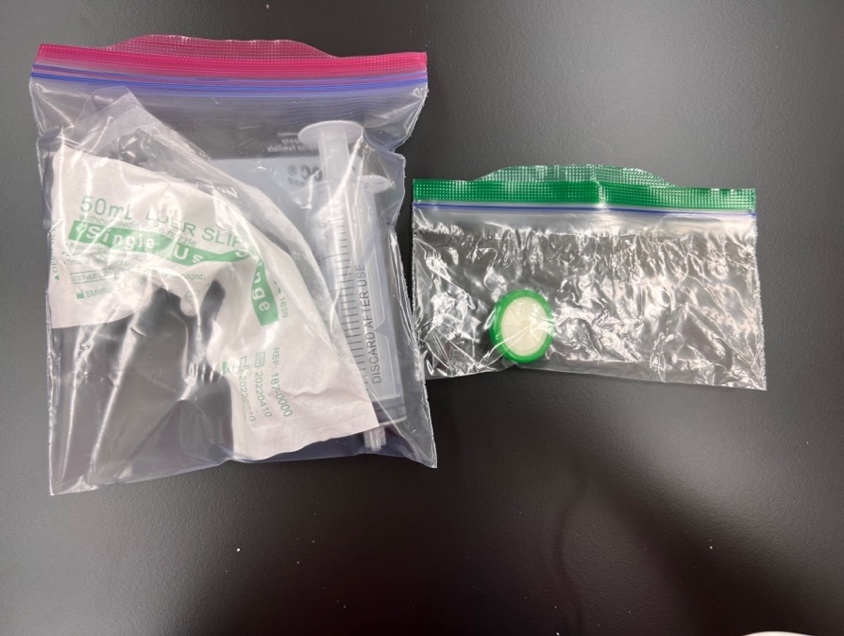


In parallel, any observable larvae will be collected using a standard larval dipper and reared to adulthood under standard insectary conditions (~27°C, relative humidity ~80%,12:12 hour light:dark cycles); if larval mortality is encountered, then these specimens will be stored for molecular analysis.

Collection of data related to sampling:

At each potential aquatic breeding site, local environmental variables will be collected using an electronic questionnaire (Study Site Questionnaire). This includes information on site ecology, approximate water volume/depth, temperature, pH, total dissolved solids (TDS), electrical conductivity (EC) (using a water quality tester), velocity, and visual co-occupancy (e.g., with other insects or tadpoles). A water probe should be used to obtain water sample variables. Try to collect as much data as possible.

At each water sample site, global positioning system (GPS) coordinates, elevation, ambient temperature and humidity will be recorded using study investigators’ smart phones. Print a few copies of the form and fill the paper forms when working in areas with bad/no internet. Re-enter the data into the online form as soon as possible. This will automatically populate the online database.

Storage of samples from re-usable filters:

Once back in the lab, prepare the following:

- Filter samples
- 15ml or 50ml falcon tubes or bijou tubes (anything with a volume of ~10ml will work)
- Forceps
- Ethanol (70% v/v)
- Bleach (10% v/v)
- Paper towel
- Marker pen

1. Wipe forceps with ethanol to make sure they are clean.
2. Disassemble the filter, making sure not to lose any of the parts.
3. Pick up the filter with forceps and place it into the tube. Sterilise the forceps with ethanol when handling different samples.
4. Label the tube with eDNA sample number (from the database), location and date.
5. Place the used filter into its ziplock bag.
6. Store the samples in the freezer (-20^o^C) until further processing.

Cleaning of re-usable filters:

1. Disassemble all the parts of the filter holder. Some of the parts such as the support grid disc are small and almost transparent, and can be easily lost, so do this carefully.
2. Wash using deionized water. If any bits of dirt or algae get stuck in the filter, use a toothbrush or a toothpick/hair pin to gently prize out.
3. Use cups or a tray and place all the parts of all the filters into bleach. Leave in bleach overnight.
4. Remove bleach and pour over first with 70% ethanol and then boiling deionized water.
5. Remove all the separate filter holder pieces and air dry on clean paper towels.
6. Assemble in the lab with new filters before the next filed trip.

Water sample collection if filters are not available but sample collection should take place:

You will need sterile 50ml Falcon tubes and something to collect water with.

1. Three replicates of ~50ml of water will be removed from the source and used to fill three 50ml Falcon tubes.
2. Label each sample with: name of location (e.g. Nima #2, Teshie #1, etc.), date of collection, number of sample (e.g. eDNA 6).
3. Store at 4^o^C (in fridge or cool box) if possible or at room temperature until you return to the lab.
4. As soon as you return to the lab, freeze the water samples immediately. They should not be defrosted until you are able to filter them.
5. At each potential aquatic breeding site, local environmental variables will be collected using an electronic questionnaire (Study Site Questionnaire), as described above.
6. In parallel, any observable larvae will be collected using a standard larval dipper and reared to adulthood under standard insectary conditions (~27°C, relative humidity ~80%,12:12 hour light:dark cycles); if larval mortality is encountered, then these specimens will be stored for molecular analysis.

Once filters are available, use the procedures described above. Make sure all the samples are labelled appropriately.

**Supplementary Method 2.** **Standard Operating Procedure for eDNA extraction**

You will need ZymoBIOMICS DNA Microprep Kit (<https://zymoresearch.eu/products/zymobiomics-dna-microprep-kit>). The eDNA extraction protocol has been slightly modified from the original Zymo protocol.

- 1. *Preparation*

1. Prepare an eDNA extraction control by placing a sterile filter inside of a 15ml or 50ml Falcon tube, and process in parallel, as below.
   1. *eDNA extraction*
2. Add sample to a **ZR BashingBead™ Lysis Tube** – simply scrunch the filter into the tube but not into a tight ball or cut it into pieces (the entire filter fits into one tube).
3. Add 750 µl **ZymoBIOMICS™ Lysis Solution** to the tube and cap tightly.
4. Secure in a bead beater fitted with a 2 ml tube holder assembly and process using optimized beat beating conditions (speed and time) for your device.

ALTERNATIVE if bead beater is not available vortex each tube for 5 min.

1. Centrifuge the **ZR BashingBead™ Lysis Tubes** in a microcentrifuge at ≥ 8,000 x g for 1 minute.
2. Transfer up to 700 µl supernatant to the **Zymo-Spin™ III-F Filter** in a Collection Tube and centrifuge at 12,000 x g for 1 minute. Discard the Zymo-Spin™ III-F Filter.
3. Add 1,000 µl of **ZymoBIOMICS™ DNA Binding Buffer** to the filtrate in the Collection Tube from Step 4. Mix well.
4. Transfer 800 µl of the mixture from Step 5 to a **Zymo-Spin™ IC Column** in a Collection Tube and centrifuge at 8,000 x g for 1 minute.
5. Discard the flow through from the Collection Tube and repeat Step 6.
6. Add 200 µl **ZymoBIOMICS™ DNA Wash Buffer 1** to the ZymoSpin™ IC Column in a new Collection Tube and centrifuge at 10,000 x g for 1 minute. Discard the flow-through.
7. Add 500 µl **ZymoBIOMICS™ DNA Wash Buffer 2** to the **ZymoSpin™ IC Column** in a Collection Tube and centrifuge at 8,000 x g for 1 minute. Discard the flow-through.
8. Add 200 µl **ZymoBIOMICS™ DNA Wash Buffer 2** to the **ZymoSpin™ IC Column** in a Collection Tube and centrifuge at 10,000 x g for 1 minute.
9. Transfer the Zymo-Spin™ IC Column to a clean 1.5 ml microcentrifuge tube and add 20 µl **ZymoBIOMICS™ DNase/RNase Free Water** directly to the column matrix and incubate for 5 minutes. Centrifuge at 10,000 x g for 1 minute to elute the DNA.
10. Place a **Zymo-Spin™ II-μHRC Filter** in a new Collection Tube and add 600 µl **ZymoBIOMICS™ HRC Prep Solution** (it dissolves the matrix in the spin column). Centrifuge at 8,000 x g for 3 minutes.
11. Transfer the eluted DNA (Step 11) to a prepared **Zymo-Spin™ II-μHRC Filter** in a clean 1.5 ml microcentrifuge tube and centrifuge at exactly 16,000 x g for 3 minutes.

The filtered eDNA is now suitable for PCR and other downstream applications.

**Supplementary Table 3.** Target amplicons covering key insecticide resistance and species-specific SNPs (AgamP4 reference genome).

| **Chr** | **Start Position** | **End Position** | **Gene Target** |
| --- | --- | --- | --- |
| **2L** | 2390813 | 2391328 | VGSC1_D1 |
| **2L** | 2422417 | 2422919 | VGSC1_D2 |
| **2L** | 2429356 | 2429845 | VGSC1_D3 |
| **2L** | 2430093 | 2430593 | VGSC1_D4 |
| **2R** | 3491732 | 3492229 | ACE1 |
| **3R** | 28597778 | 28598182 | GSTE2 |
| **2L** | 25428861 | 25429373 | RDL |
| **Mt** | 1424 | 2960 | COX1 |
| **UNKN** | 36384024 | 36384413 | IGS |
| **X** | 22951331 | 22951809 | SINE200 |
| **UNKN** | 31004850 | 31005267 | ITS1 |
| **UNKN** | 35962837 | 35963328 | ITS2 |
| **Mt** | 8308 | 8824 | MtND4 |

**Supplementary Table 4.** Primers for insecticide resistance amplicon sequencing for the *Anopheles gambiae* s.l. complex.

| **Target Gene** | **Amplicon** | **Forward Primer** | **Reverse Primer** | **Chromosome** | **Position*** | **Product Size (bp)*** |
| --- | --- | --- | --- | --- | --- | --- |
| *vgsc* | VGSC-I | ATTCGTTATTCTTCAGATGAACT | ATTCTCACCCGAAGTGC | AgamP4_2L | 2390813-2391328 | 517 |
|  | VGSC-II | GTTTTGCTAGCCTAATTGC | TGTCGGTTGAACGGATGCTATT | AgamP4_2L | 2422417-2422919 | 503 |
|  | VGSC-III | TTCATGGGAAAATTCACCAA | AATTAGTGCTCCAAACACAAAC | AgamP4_2L | 2429356-2429845 | 490 |
|  | VGSC-IV | CGAGCCATGGAATTTGT | TGATGTGATCCAGTTACAGA | AgamP4_2L | 2430093-2430593 | 501 |
| *ace-1* | ACE1 | CTGGTGGTCAACACGGA | GAACAGTCCCGCATTGC | AgamP4_2R | 3491732-3492229 | 498 |
| *gste2* | GSTE2 | GCCCGGATGAGATTCAT | GGCTAGCACAAACTTGC | AgamP4_3R | 28597778 -28598182- | 404 |
| *rdl* | RDL | CATTGCAATCATCACCATCA | CCAGCAGACTGGCAAATACC | AgamP4_2L | 25428861-25429373 | 500 |

*Position and product size in *An. gambiae* AgamP4 reference genome*.*

**Supplementary Table 5.** Target SNPs, which have been associated with insecticide resistance in the *Culicidae* family. Genomic positions according to the AgamP4 reference genome.

| **Gene** | **Chromosome** | **Position** | **SNP** | **Amino Acid Change** |
| --- | --- | --- | --- | --- |
| VGSC (DI-S6) | AgamP4_2L | 2391228 | G>C, T | V402L |
| VGSC (DII-S6) | AgamP4_2L | 2422575 | T >C | S970P |
| VGSC (DII-S6) | AgamP4_2L | 2422643 | A>G | I992M |
| VGSC (DII-S6) | AgamP4_2L | 2422651 | T >C | L995S |
| VGSC (DII-S6) | AgamP4_2L | 2422652 | A>T | L995F |
| VGSC (DII-S6) | AgamP4_2L | 2422657 | T>G | V997G |
| VGSC (DIII-S6) | AgamP4_2L | 2429617 | T>C | I1527T |
| VGSC (DIII-S6) | AgamP4_2L | 2429623 | T>G | F1529C |
| VGSC (DIII-S6) | AgamP4_2L | 2429745 | A>T | N1570Y |
| VGSC (DIV-S5) | AgamP4_2L | 2430424 | G>T | A1746S |
| VGSC (DIV-S5) | AgamP4_2L | 2430460 | G>T | D1758Y |
| GSTE2 | AgamP4_3R | 28598166 | T>C | I114T |
| GSTE2 | AgamP4_3R | 28598062 | C>G | L119V |
| GSTE2 | AgamP4_3R | 28598057 | C>G,A | F120L |
| RDL | AgamP4_2L | 25429236 | C>G | A296G |
| RDL | AgamP4_2L | 25429235 | G>T | A296S |
| ACE1 | AgamP4_2R | 3492074 | G>A | G280S |

**Supplementary Data 1**. All quality filtered SNPs are included in the supplementary excel file (Supplementary_Data_combined_genotyped_filtered_formatted.snps.trans).
